# The two redox states of the human NEET proteins’ [2Fe–2S] clusters

**DOI:** 10.1007/s00775-021-01890-8

**Published:** 2021-08-28

**Authors:** Ke Zuo, Henri-Baptiste Marjault, Kara L. Bren, Giulia Rossetti, Rachel Nechushtai, Paolo Carloni

**Affiliations:** 1 The Alexander Silberman Institute of Life Science, The Hebrew University of Jerusalem, Edmond J. Safra Campus at Givat Ram, 91904 Jerusalem, Israel; 2 Department of Physics, RWTH Aachen University, 52074 Aachen, Germany; 3 Department of Chemistry, University of Rochester, Rochester, NY 14627-0216 USA; 4 Computational Biomedicine, Institute of Advanced Simulation IAS-5 and Institute of Neuroscience and Medicine INM-9, Forschungszentrum Jülich GmbH, 52425 Jülich, Germany; 5 Jülich Supercomputing Center (JSC), Forschungszentrum Jülich GmbH, Jülich, Germany; 6 Department of Neurology, Faculty of Medicine, RWTH Aachen University, 52074 Aachen, Germany; 7 JARA Institute: Molecular Neuroscience and Imaging, Institute of Neuroscience and Medicine INM-11, Forschungszentrum Jülich GmbH, 52425 Jülich, Germany

**Keywords:** Iron–sulfur cluster lability, Ab initio calculations, Kinetic measurements, Human NEET proteins, Evolutionary analysis

## Abstract

The NEET proteins constitute a unique class of [2Fe–2S] proteins. The metal ions bind to three cysteines and one histidine. The proteins’ clusters exist in two redox states; the oxidized protein (containing two Fe^III^ ions) can transfer the cluster to apo-acceptor protein(s), while the reduced form (containing one ferrous ion) remains bound to the protein frame. Here, we perform in silico and in vitro studies on human NEET proteins in both reduced and oxidized forms. Quantum chemical calculations on all available human NEET proteins structures suggest that reducing the cluster weakens the Fe–N^His^ and Fe–S^Cys^ bonds, similar to what is seen in other Fe–S proteins (e.g., ferredoxin and Rieske protein). We further show that the extra electron in the [2Fe–2S]^+^ clusters of one of the NEET proteins (mNT) is localized on the His-bound iron ion, consistently with our previous spectroscopic studies. Kinetic measurements demonstrate that the mNT [2Fe–2S]^+^ is released only by an increase in temperature. Thus, the reduced state of human NEET proteins [2Fe–2S] cluster is kinetically inert. This previously unrecognized kinetic inertness of the reduced state, along with the reactivity of the oxidized state, is unique across all [2Fe–2S] proteins. Finally, using a coevolutionary analysis, along with molecular dynamics simulations, we provide insight on the observed allostery between the loop L2 and the cluster region. Specifically, we show that W75, R76, K78, K79, F82 and G85 in the latter region share similar allosteric characteristics in both redox states.

**Graphic abstract:**

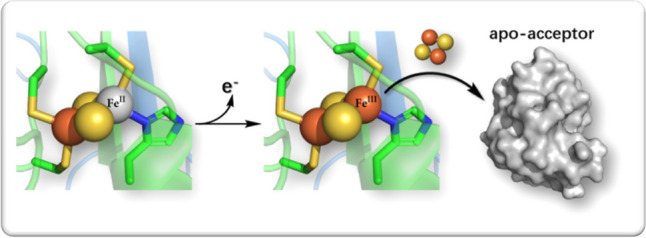

**Supplementary information:**

The online version contains supplementary material available at 10.1007/s00775-021-01890-8.

## Introduction

**Chart 1:**
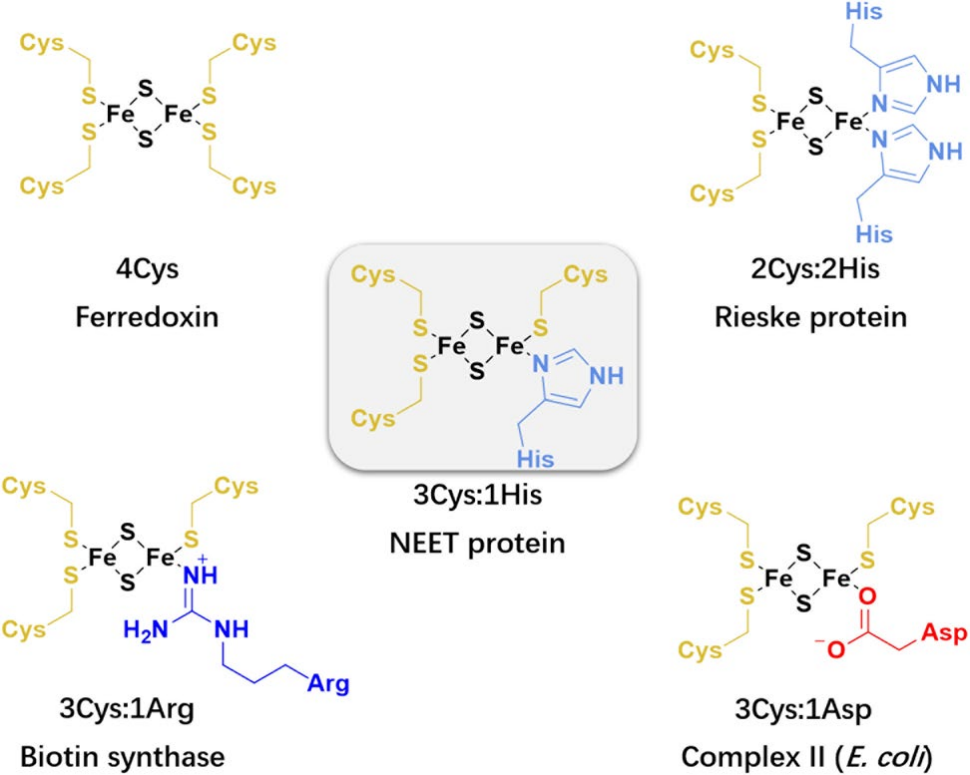
Metals’ coordination in [2Fe–2S] proteins

Two iron–two sulfur [2Fe–2S] proteins perform electron transfer, serve as oxygen/iron sensors and transcription factors, and perform enzymatic reactions and many other functions across the three kingdoms of life [1, 2]. They contain a ferrous and ferric or two ferric ions in their reduced and oxidized forms, respectively [3]. The metal ions bind usually to four Cys residues. Yet, in few cases, Asp, Arg and/or His residues [4] replace one or two cysteines (Chart 1). Among these are the NEET proteins, featuring two clusters with 3Cys:1His coordination (Chart 1) [5].

Three human NEET proteins have been identified. One (MiNT) is located only inside the mitochondria [6]. The other two, mNT and NAF-1 are anchored to the outer mitochondrial membrane with NAF-1 also present on the surface of the endoplasmic reticulum and its mitochondrial associated membrane [6]. Under healthy conditions [7], they are usually in their reduced, dormant state [8, 9], because of their reducing environment. In the oxidized state, often triggered by oxidative stress [6], the human NEET proteins are able to transfer their [2Fe–2S] clusters to apo-acceptor proteins without the aid of specific chaperons [10] such as the mitochondrial human GLRX5 [11, 12] and the cytosolic human GLRX3 [13] [2Fe–2S] proteins. Unfortunately, aberrant cluster transfer upon oxidative conditions can occur during pathological conditions as in cancer, metabolic and neurodegenerative diseases [6]. Hence, human NEET proteins are promising targets for treating a variety of diseases, from cancer to neurodegenerative diseases [6].

The electronic and structural properties of human oxidized NEET proteins [14, 15], along with their kinetic properties [10, 16], have been characterized. Here, by performing density functional theory (DFT) calculations, molecular dynamics (MD) simulations and in vitro experiments on the reduced and oxidized forms, along with a coevolutionary analysis of these proteins, we provide insight on the reduced state and offer a detailed comparison between the two redox states of the human NEET proteins.

## Results and discussion

**Fig. 1:**
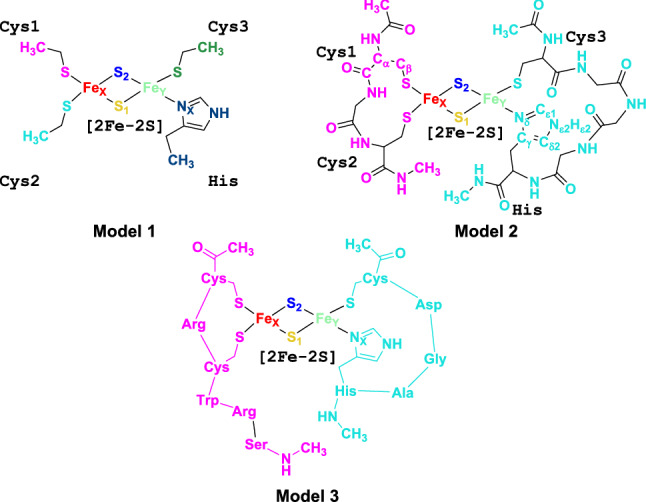
Schematic of models **1**, **2** and **3** used for the QM calculations presented in this work. In **1**, **2** and **3**, the [2Fe–2S] clusters are separated into four independent fragments as in Ref. [17] (Fe_X_, Fe_Y_, S_1_ and S_2_ in the figure). The ligation residues are treated as separate four, two and two fragments in **1**, **2** and **3**, respectively [17]. The fragments are colored differently

Our investigation is carried out in three steps. First, we perform DFT calculations on three models of the reduced metal sites (Fig. 1) to investigate their electronic properties. This work builds on our previous DFT investigations of the oxidized state [17] and it presents also new results for the latter. Next, we perform kinetic measurements to investigate the lability of the reduced cluster at different temperatures. Finally, we carry out a coevolutionary analysis, based on molecular dynamics (MD) simulations on the oxidized and reduced forms performed in this work, to provide insight on the identified allostery between a loop opposed to the cluster (L2 in Fig. 5C) and the cluster binding region [18].

### Quantum chemistry

Calculations at the B3LYP/6–311++G(2d,2p) [19, 20] level of theory (already used for Fe–S proteins [21], including oxidized human NEET proteins [17]) are carried out on all of the 12 human NEET PDB structures (Table S1 and Fig. 1), in both the reduced and oxidized states. These are ten mNT and two NAF-1 proteins. Some of results for the oxidized state have been already reported in Ref. [17].

**Fig. 2:**
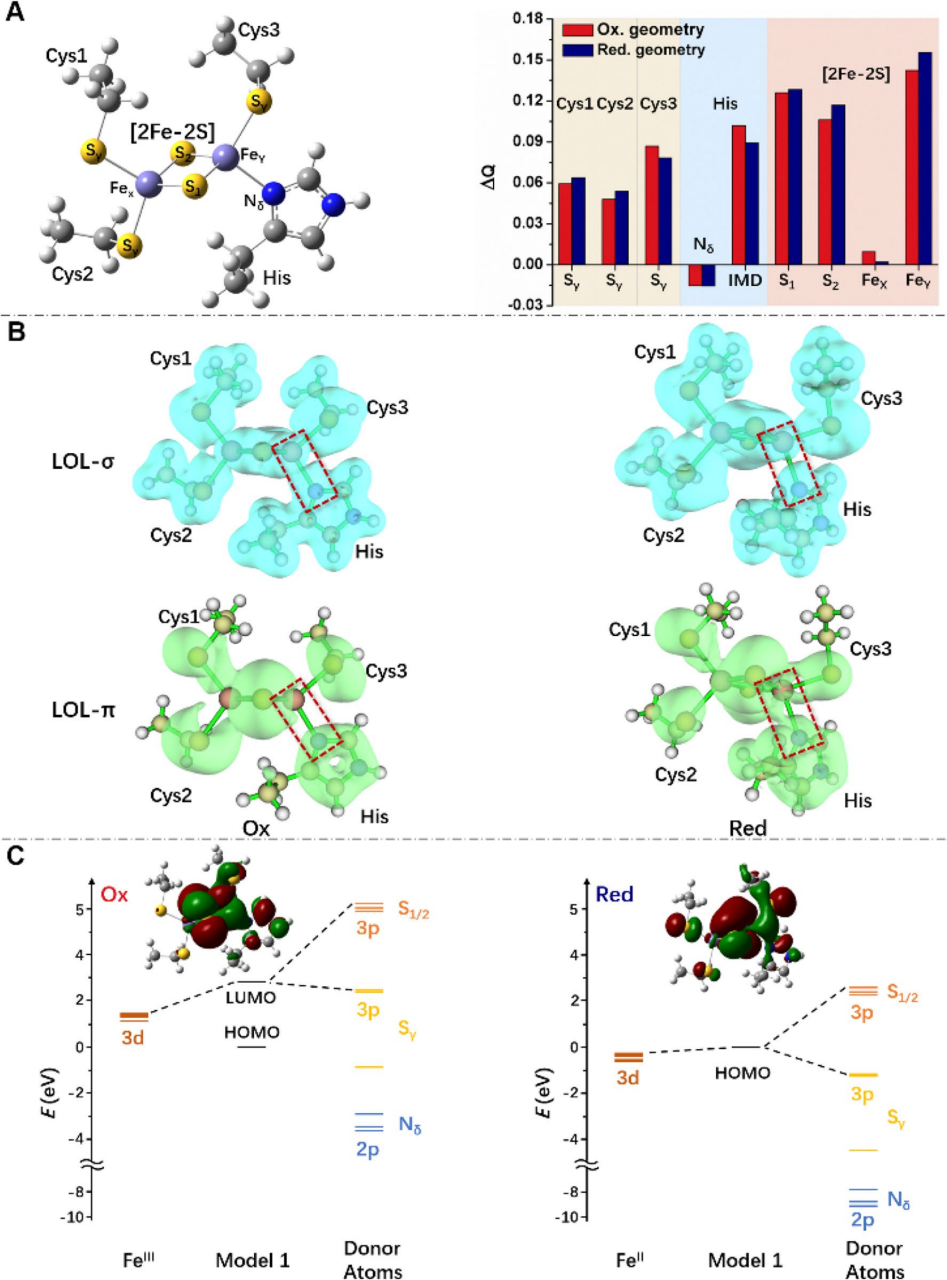
The two redox states of the clusters in human NEET proteins. **A** The charge (right) upon reduction of the oxidized state is localized to His-bound iron Fe_Y_, bridge sulfur S_1_ and S_2_, and S_γ_ of Cys ligands while it decreases on N_δ_ of His ligand. **B** Contours (0.2 e/Å^3^) of the localized orbital locator (LOL) [30] for the complex and in particular for the histidine-iron σ and π bonds. **C** The molecular orbitals involved in the reduction are the LUMO of the oxidized form and the HOMO of the reduced form, as emerging from an analysis of the Kohn–Sham orbitals. Both involve the surface localized His-bound iron and not the other iron. The HOMO energy is set to zero for each state. Contours shown at 0.01 e/Å^3^. The calculations are carried out at the B3LYP/6-311++G(2d,2p) [19, 20] level of theory for the PDB ID: 2QH7 structure [22]. Very similar results are obtained for all the other 11 structures analyzed here and for calculations at the B(5%HF)P86 [23, 24] level of theory (data not shown)

The calculations shed light on the strength of the Fe–N^His^ and Fe–S^Cys^ bonds upon reduction: the force constants and delocalization indexes, which correlate with bond strengths and bond orders [25], respectively, decrease (Tables 1, 2, and S3). The Fe-bound His is considered here to be protonated (Fig. 2A). Similar conclusions are drawn: (i) when considering the His to be deprotonated (Table 2); (ii) when including the protein frame’s electric field and thermal fluctuations of the protein using results from MD simulations performed in this work (see “Methods”); (iii) when employing another functional used for Fe–S proteins [B(5%HF)P86/def–TZVP] [23, 24, 26]; (iv) when including dispersion contributions, which can be significant in DFT calculations on proteins [27]. Thus, these results appear to be quite robust. They are fully consistent with previous studies which point to a decrease of iron-donor atom bond strength upon the reduction of 4Cys-coordinated [28] and 2Cys:2His [2Fe–2S] proteins [29].

**Table 1:** Average bond lengths (*R*_min_, in nm) and bond force constants (*K*_r_, in kJ/mol nm^2^) of [2Fe–2S] clusters as obtained by geometry optimizations of model **1**, based on the human mNT structure (PDB ID: 2QH7^22^)

Bond	*R*_min_	*K*_r_	Bond	*R*_min_	*K*_r_
B3LYP, with protonated Fe-bound His (RMSD = 0.004/*0.014* nm)					
Fe_X_–S_1_	**0.228**	**31,254.5**	Fe_X_–S^Cys1^	**0.232**	**33,555.7**
Fe_X_–S_2_	**0.229**	**29,497.2**	Fe_X_–S^Cys2^	**0.232**	**33,095.4**
Fe_Y_–S_1_	**0.221**	**46,358.7**	Fe_Y_–S^Cys3^	**0.233**	**31,882.1**
Fe_Y_–S_2_	**0.221**	**45,145.4**	Fe_Y_–N^His^	**0.218**	**16,359.4**
Fe_X_–S_1_	*0.226*	*36,024.2*	Fe_X_–S^Cys1^	*0.240*	*23,472.2*
Fe_X_–S_2_	*0.227*	*35,187.4*	Fe_X_–S^Cys2^	*0.240*	*23,263.0*
Fe_Y_–S_1_	*0.230*	*30,585.0*	Fe_Y_–S^Cys3^	*0.242*	*17,572.8*
Fe_Y_–S_2_	*0.231*	*29,078.8*	Fe_Y_–N^His^	*0.230*	*7740.4*
B3LYP, with unprotonated Fe-bound His (RMSD = 0.007/*0.006* nm)					
Fe_X_–S_1_	**0.227**	**34,267.0**	Fe_X_–S^Cys1^	**0.236**	**27,154.2**
Fe_X_–S_2_	**0.226**	**34,225.1**	Fe_X_–S^Cys2^	**0.234**	**29,999.3**
Fe_Y_–S_1_	**0.225**	**35,982.4**	Fe_Y_–S^Cys3^	**0.236**	**27,196.0**
Fe_Y_–S_2_	**0.226**	**35,522.2**	Fe_Y_–N^His^	**0.205**	**32,300.5**
Fe_X_–S_1_	*0.225*	*39,413.3*	Fe_X_–S^Cys1^	*0.246*	*17,028.9*
Fe_X_–S_2_	*0.225*	*38,534.6*	Fe_X_–S^Cys2^	*0.243*	*19,706.6*
Fe_Y_–S_1_	*0.237*	*21,212.9*	Fe_Y_–S^Cys3^	*0.246*	*13,012.2*
Fe_Y_–S_2_	*0.237*	*20,878.2*	Fe_Y_–N^His^	*0.217*	*14,644.0*
B(5%HF)P86, with protonated Fe-bound His (RMSD = 0.005/*0.017* nm)					
Fe_X_–S_1_	**0.224**	**32,384.2**	Fe_X_–S^Cys1^	**0.230**	**31,923.9**
Fe_X_–S_2_	**0.225**	**30,961.6**	Fe_X_–S^Cys2^	**0.231**	**31,338.2**
Fe_Y_–S_1_	**0.219**	**45,563.8**	Fe_Y_–S^Cys3^	**0.231**	**31,589.2**
Fe_Y_–S_2_	**0.219**	**43,890.2**	Fe_Y_–N^His^	**0.216**	**15,020.6**
Fe_X_–S_1_	*0.225*	*36,191.6*	Fe_X_–S^Cys1^	*0.238*	*24,016.2*
Fe_X_–S_2_	*0.225*	*35,689.5*	Fe_X_–S^Cys2^	*0.237*	*23,848.8*
Fe_Y_–S_1_	*0.224*	*35,438.5*	Fe_Y_–S^Cys3^	*0.236*	*20,836.3*
Fe_Y_–S_2_	*0.225*	*32,969.9*	Fe_Y_–N^His^	*0.223*	*5230.0*
B(5%HF)P86, with unprotonated Fe-bound His (RMSD = 0.008/*0.007* nm)					
Fe_X_–S_1_	**0.223**	**36,442.6**	Fe_X_–S^Cys1^	**0.235**	**27,279.7**
Fe_X_–S_2_	**0.223**	**36,861.0**	Fe_X_–S^Cys2^	**0.232**	**30,375.8**
Fe_Y_–S_1_	**0.223**	**37,363.1**	Fe_Y_–S^Cys3^	**0.235**	**26,986.8**
Fe_Y_–S_2_	**0.224**	**36,484.5**	Fe_Y_–N^His^	**0.204**	**31,212.6**
Fe_X_–S_1_	*0.224*	*38,785.7*	Fe_X_–S^Cys1^	*0.245*	*17,321.8*
Fe_X_–S_2_	*0.224*	*38,409.1*	Fe_X_–S^Cys2^	*0.240*	*20,459.8*
Fe_Y_–S_1_	*0.230*	*25,355.0*	Fe_Y_–S^Cys3^	*0.240*	*15,522.6*
Fe_Y_–S_2_	*0.231*	*24,685.6*	Fe_Y_–N^His^	*0.215*	*14,309.3*

As mentioned above, the geometry optimizations of the 12 X-ray structures lead to very similar structural determinants. Thus, only the results for one of them (PDB ID: 2QH7 [22]) are reported here. The calculations are carried out at the B3LYP/6-311++G(2d,2p) [19, 20] (top) and B(5%HF)P86/def-TZVP [23, 24] (bottom) levels. Both the oxidized (in bold face) and reduced (in italics) states are considered. The protonation state of the iron-bound histidine is specified in the table. The overall RMSD relative to the initial structures are reported in parentheses

**Table 2:** Delocalization indexes of the Fe–N^His^ and Fe–S bond in oxidized (top line, bold face) and reduced (bottom line, italics) states, respectively

	Fe_X_–S^Cys1^	Fe_X_–S^Cys2^	Fe_X_–S_1_	Fe_X_–S_2_	Fe_Y_–S^Cys3^	Fe_Y_–N^His^	Fe_Y_–S_1_	Fe_Y_–S_2_
B3LYP geometry	Protonated His							
	** 0.567**	**0.553**	**0.610**	**0.590**	**0.544**	**0.281**	**0.755**	**0.736**
	* 0.476*	*0.471*	*0.685*	*0.677*	*0.457*	*0.234*	*0.579*	*0.557*
	** 0.583**^**†**^	**0.564**^**†**^	**0.603**^**†**^	**0.602**^**†**^	**0.557**^**†**^	**0.306**^**†**^	**0.742**^**†**^	**0.745**^**†**^
	* 0.491*^**†**^	*0.486*^**†**^	*0.686*^**†**^	*0.672*^**†**^	*0.466*^**†**^	*0.269*^**†**^	*0.575*^**†**^	*0.560*^**†**^
	Deprotonated His							
	** 0.524**^**†**^	**0.533**^**†**^	**0.651**^**†**^	**0.647**^**†**^	**0.522**^**†**^	**0.410**^**†**^	**0.658**^**†**^	**0.648**^**†**^
	* 0.442*^**†**^	*0.459*^**†**^	*0.721*^**†**^	*0.709*^**†**^	*0.440*^**†**^	*0.353*^**†**^	*0.506*^**†**^	*0.497*^**†**^
B(5%HF)P86 geometry	Protonated His							
	** 0.633**	**0.579**	**0.730**	**0.734**	**0.570**	**0.426**	**0.740**	**0.723**
	* 0.482*	*0.483*	*0.694*	*0.687*	*0.484*	*0.257*	*0.619*	*0.590*
	**0.562**	**0.563**	**0.648**	**0.625**	**0.542**	**0.296**	**0.754**	**0.745**
	*0.475*	*0.491*	*0.704*	*0.668*	*0.473*	*0.275*	*0.599*	*0.612*
	Deprotonated His							
	** 0.516**	**0.537**	**0.659**	**0.665**	**0.515**	**0.403**	**0.682**	**0.668**
	* 0.425*	*0.454*	*0.729*	*0.720*	*0.451*	*0.335*	*0.542*	*0.528*
	**0.515**	**0.531**	**0.680**	**0.658**	**0.514**	**0.398**	**0.675**	**0.664**
	*0.429*	*0.458*	*0.737*	*0.705*	*0.448*	*0.327*	*0.544*	*0.535*

These have been obtained by unrestricted HF/6-311++G(2d,2p) calculations on model **1** as in Ref. [25], based on the B3LYP/6-311++G(2d,2p) [19, 20] and B(5%HF)P86/def-TZVP [23, 24] optimized structures. The geometry optimizations are carried out either without constraints or with constraints on the C_α_ atoms (underscored number). Numbers with a dagger symbol were obtained by adding DFT-D3(BJ) corrections [27, 31, 32] in geometry optimizations (see “Methods”). Because the latter are almost identical across the structures investigated here, the results of only one of them (Human mNT, PDB ID: 2QH7 [22]) are reported

Our QM calculations provide information also into the change in charge distribution in the cluster. The extra charge density turns out to involve the His-bound iron ion, close to the protein surface, and its ligands (Fig. 2A). Indeed, the largest increase in electronic charge is mainly localized on that iron ion, on the two inorganic sulfurs and the histidine. Interestingly, however, the nitrogen donor atom decreases its charge. This is likely to be caused by a competition between the Fe–N^His^ σ bond, which may be stronger in the oxidized state [33], and the π bond involving the aromatic ring and the metal ion. An analysis of the localized orbital locator (LOL) values on the critical points, which correlate with bond strength [30], suggests that this is the case. Indeed, on passing from the oxidized to the reduced forms, the LOL σ values decrease from 0.21 to 0.19, while the LOL π values increase from 0.15 to 0.24, overcoming the contribution of the σ donation (Figs. 2B and S1). The reduction process is carried by the lowest unoccupied molecular orbital (LUMO) of the oxidized form, ready to accept the electron upon reduction, and the highest occupied MO (HOMO) of the reduced form; both are mainly localized on the His-bound iron ion, close to the protein surface, but not the other iron ion (Figs. 2C and S2–S6). We conclude that the His-bound iron, located at the surface of the protein, is the one that gets reduced. This is fully consistent with spectroscopic studies, which show that the reduced form features a ferrous iron bound to His87 [14]. We close this section by pointing out that one of the three human NEET proteins, mNT, exhibits highly complex proton coupled electron transfer processes during reduction [16], although a clear link between these processes and cluster release has not emerged yet. This process is not investigated here: we rather focus on the initial and final states of the process (i.e., four possible oxidized/reduced [2Fe–2S] with protonated/deprotonated His ligand states, see Fig. 2A).

### Kinetic measurements

**Fig. 3:**
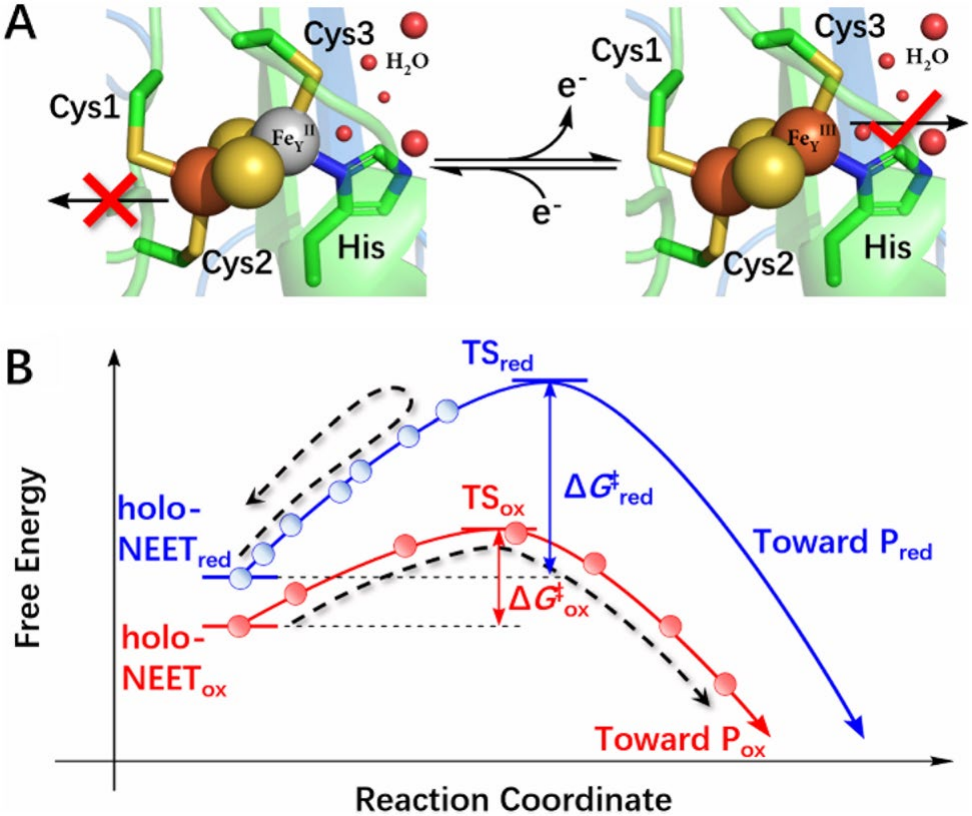
Redox-dependent lability of NEET proteins’ [2Fe–2S] clusters. **A** The reduced clusters remain stable through a large range of pH [16] (left). Oxidation allows their transfer to an apo-acceptor-protein(s) (right). **B** Proposed free energy landscape associated with the [2Fe–2S] cluster release, described by a generic Reaction Coordinate. Here, the lower free energy of the oxidized state is assumed based on our DFT calculations. TS for transition state, and P_red_ and P_ox_ for products [apo-NEET proteins along with the released cluster either to water solution [10] (in vitro) or to their cellular partners [11] (in vivo) in the reduced and oxidized forms, respectively]. The schematic is qualitative

**Fig. 4:**
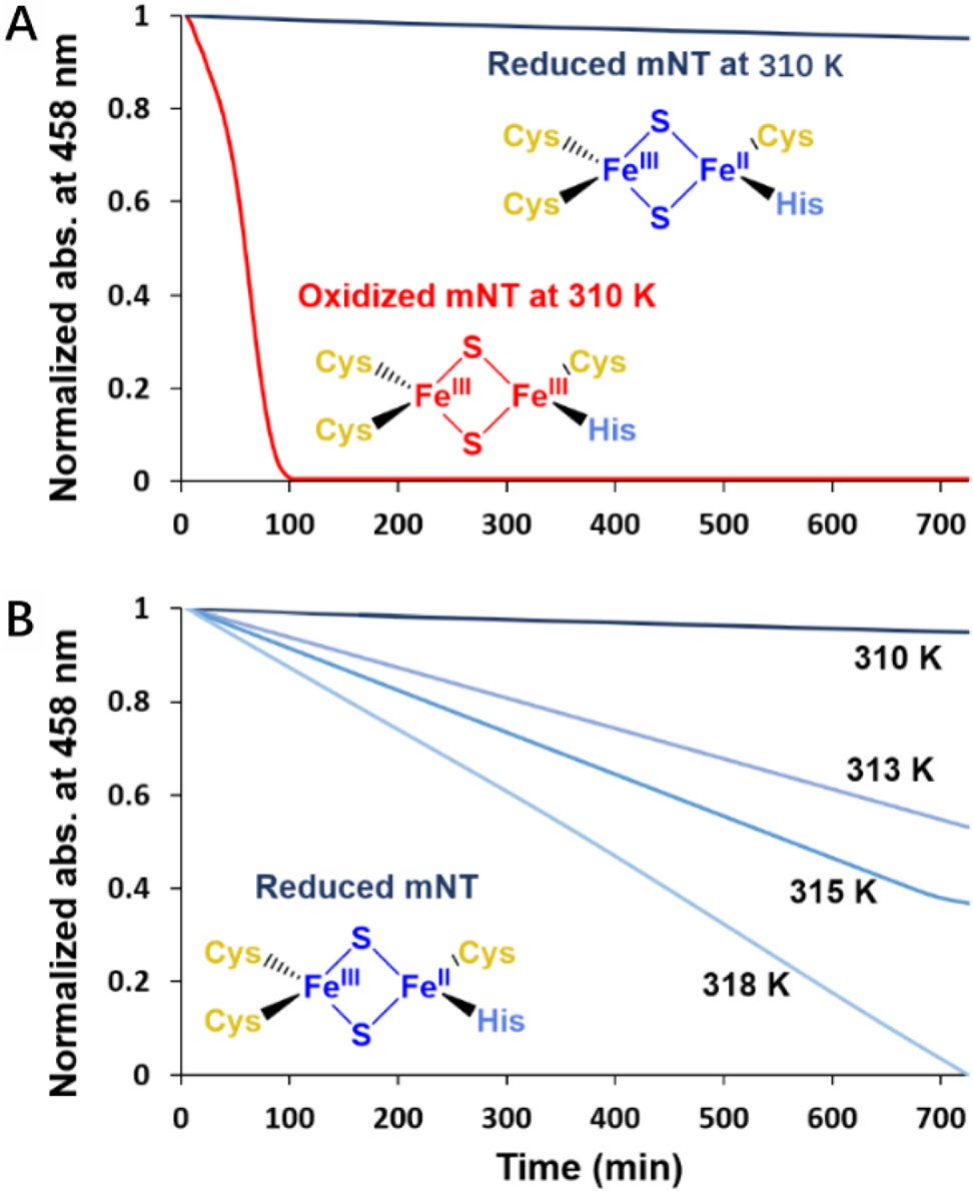
Lability of [2Fe–2S] clusters of mNT at different temperatures. The absorbance and stability of the clusters are monitored at their characteristic absorption peak of 458 nm. **A** At 310 K, the oxidized clusters are highly labile and dissociate from the protein (red line), while the reduced clusters are stable (dark blue lines). **B** By increasing the temperature up to 318 K the latter induces a loss of their stability (from dark blue to light blue lines). The measurements have been taken at pH 6.0, which is closer to the physiological pH in pathologies like cancer [34] and renders the reaction in vitro faster than that at pH 8.0

The reduced state is inert at room temperature [16]. So, we expect that the free energy barrier of cluster transfer is higher at the transition state in the reduced form (Δ*G*^‡^_red_ > Δ*G*^‡^_ox_, see Fig. 3). To test this hypothesis, we measured the in vitro stability of the [2Fe–2S] clusters of the wild type mNT protein as well as H87C variant in the reduced state at different temperatures (Figs. 4 and S7). For a proper comparison with the oxidized state, we perform the measurements for the oxidized state (already reported in Ref. [10]), in exactly the same conditions as those carried out for the reduced form. The oxidized [2Fe–2S] clusters are highly labile at 310 K, as already observed [16] (Δ*G*^‡^_ox_ ≈ *k*_B_*T*) while the reduced ones remain stable over a long time in the same conditions (Fig. 4A). Thus, here Δ*G*^‡^_red_ > *k*_B_*T*. However, the lability of the reduced correlates with the rise of temperature to 313, 315 and 318 K (Fig. 4B). Taking these results together with those of the previous section, we suggest that reduction weakens the coordination bonds but the cluster stays bound to the protein at physiological temperature because of a relatively high free energy barrier that hampers its release (Fig. 4); i.e., the reaction is kinetically controlled. On the other hand, the oxidized state, albeit more thermodynamically stable, is labile at room temperature because of the small barrier to release (Fig. 4). Interestingly, increasing the temperature turns out not affect the cluster lability of the H87C mutant, in which the Fe-bound histidine is replaced by a cysteine (Fig. S7). Thus, the His-Fe bound is a key determinant for the observed reactivity of the cluster at higher temperature.

### Coevolutionary analysis and molecular dynamics simulations

So far, our attention has focused on specific properties of the cluster, from the electronic structure to kinetics. We close this section by investigating an important property involving the *entire* protein, in both the reduced and oxidized states. This is the allostery between L2 present in mNT’s β-cap domain and the cluster binding region, which was observed by one of us many years ago [35]. This allostery plays an essential role in the cluster release/transfer to apo-acceptor proteins [8, 35]. Here, we identify the specific residues involved in the allostery as well as the dependence of the latter on the redox state of the protein. Both features are currently not known.

Here, we exploit the fact that allosteric communication between different protein regions can be mirrored by pairs of coevolved residues [18]. These residues exhibit concordant patterns of evolution: they jointly mutated with a frequency higher than the average [36]. A pair of residues is considered to be strongly coevolving if the residues are not in close proximity of each other and if their coevolution score (here calculated with the CoeViz web server (http://polyview.cchmc.org/) [18] is greater than 0.7. The calculation of the co-evolution score is based on proteins’ sequences [18]. To determine the contiguity between the residue pairs, we need structural information on the reduced and oxidized mNT. Because the experimental structure of the reduced form is not available [37], here we use representative structures from 1 μs long, AMBER-based MD simulations of the oxidized and reduced mNT in solution (see SI, Section III). The simulations are based on the X-ray structure of mNT (PDB ID: 2QH7 [22]). They use an AMBER-compatible force fields developed here and in Ref. [17] for the reduced and oxidized metal clusters, respectively.

**Fig. 5:**
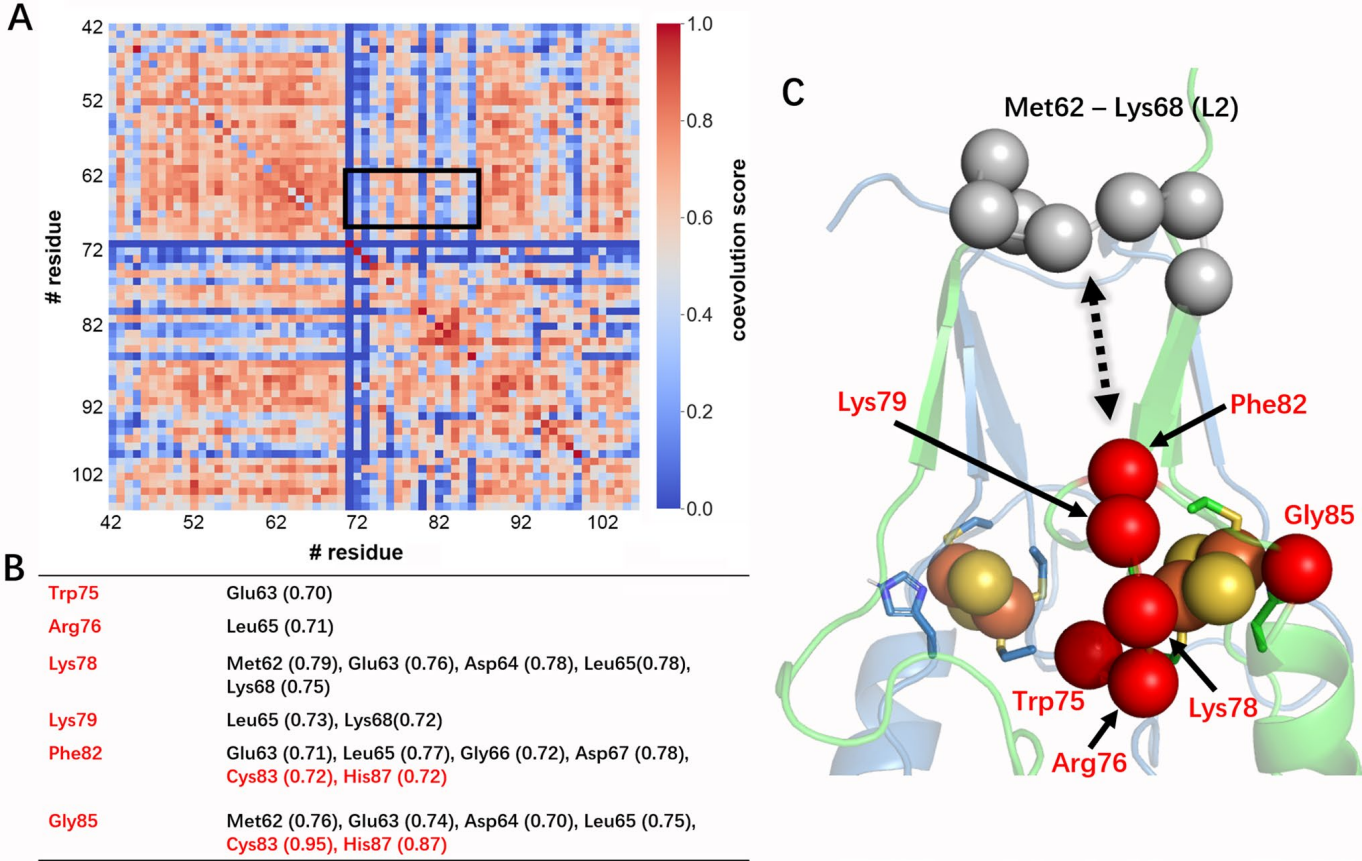
Allostery between [2Fe–2S] cluster area and L2 region of the β-cap domain of mNT. **A** The coevolution score heatmap, as obtained by CoeViz [18]. The one in the reduced state is shown in Fig. S12. **B, C** Strongly coevolving amino acid pairs from the cluster area and L2. **B** reports the coevolution scores. **C** shows the location of these residues in oxidized mNT in aqueous solution, as emerging from MD simulation (see SI, Section III). The correspondent structure for the reduced state in solution is shown in Fig. S10

The identified strong coevolving pairs are the same for both redox states (Fig. S12), consistent with the high similarity between reduced and oxidized mNT representative MD structures: the RMSD on the C_α_ atoms is as low as 0.5 Å (see Fig. S10). In particular, Trp75, Arg76, Lys78 and Lys79 adjacent to cluster binding area, along with Phe82 and Gly85 located beside ligand Cys83 and His87, turn out to strongly co-evolve with Met62–Lys68 in L2 region, and, the latter two, also with Cys83 and His87 (Figs. 5 and S12).^1^ Thus, our analysis confirms previous findings [35], and details which residues are involved in the allosteric communication. Our analysis shows that the cross-talk between the β-cap and cluster binding region of human mNT involves the same amino acid residues in oxidized and in the reduced form. However, the allosteric effect on these amino acids can differ significantly on passing from one oxidation state to the other. These findings do not provide insights into the role that allostery plays in the cluster release/transfer to apo-acceptor proteins or into the redox dependence of cluster release/transfer.

## Conclusions

We have provided here a detailed picture of the reduced form of human NEET proteins and compared it with that of the oxidized state. Our quantum chemical studies, performed across all structures available in the PDB, suggest that reduction of the oxidized cluster involves the iron bound to the histidine, consistent with experiment [14] and it weakens the coordination bonds of the metal ions, as seen for other iron–sulfur clusters. Nevertheless, the cluster remains bound in the reduced state because it is kinetically inert, as shown here by in vitro measurements on the mNT protein’s cluster stability. Finally, our in silico analysis across all human NEET proteins provide insight on the observed allostery cross-talk between the L2 of the β-cap domain and the cluster region [35]. The residues involved in it turn out to be Met62–Lys68, from the L2 loop and Trp75, Arg76, Lys78, Lys79, Phe82 and Gly85 from the cluster region for mNT in both redox states (Figs. 5 and S12).

## Methods

### QM calculations

The MCPB.py script [38] in the AMBER16 package [39] was employed to construct the QM truncated models **1**, **2** and **3** (Fig. 1). These are built from the 12 X-ray structures of human NEET proteins (mNT [22, 35, 40–44] and NAF-1 [45, 46], Table S1). In **1**, the [2Fe–2S] cluster and the side chains of ligated three cysteine residues and one histidine residue were included. All C_β_s are saturated with H atoms. In **2**, the whole ligation residues, as mentioned above, and the main chain atoms of the residues connecting those residues in the protein were included. The side chain of connection residues was replaced with H atoms. **3** included the [2Fe–2S] cluster and all residues within 4 Å from the cluster. Models **1** were terminated by methyl groups, those of **2** and **3** with acetyl (ACE) or *N*-methyl (NME) groups. Hydrogen atoms were added assuming standard bond lengths and angles.

The experimental spin state of the NEET proteins was given as an input in the fragment-combination method (Table S2) [47]. This method controls the generation of initial guess for the Hartree–Fock wavefunction from fragment guesses or self-consistent field method solutions, which converged the wavefunction to the desired antiferromagnetic state. It has been used for a variety of other Fe–S proteins [21], including NEET proteins in the oxidized state [17]. Geometry optimization of **1** was carried out with the unrestricted B3LYP/6-311++G(2d,2p) [19, 20] with and without Grimme-type empirical D3(BJ) dispersion corrections [27, 31, 32], and B(5%HF)P86/def-TZVP functionals [23, 24]. Both functionals have been widely used to study [2Fe–2S] and [4Fe–4S] clusters’ electronic structure [17, 21, 26].^2^ The geometrical optimization was carried out using the following convergence criterion: the maximum and RMS force on the nuclei are less than 0.00045 Hartrees/Bohr and 0.00035 Hartrees/Bohr, respectively, and the maximum and RMS nuclei displacement are less than 0.0018 and 0.0012 Å, respectively. The structural determinants of the optimized structures are basically identical and therefore the results are reported only for the mNT protein (PDB ID: 2QH7 [22]). Single point energy calculations, based on the X-ray structures, were carried out at the unrestricted B3LYP/6-311++G(2d,2p) level [19, 20] on **2** and **3**.

In some calculations, we applied constraints on the C_α_ atoms within geometry optimization to mimic protein environment in model **1**. We finally carried out single point calculations on model **1** with the geometry of cluster representatives of the MD simulations (see section below).

An in-house code (cpmd-cube-tools: https://pypi.org/project/cpmd-cube-tools/) [50] was used to calculate the change in electron density ($\Delta \rho$) upon reduction of **1**:

$$\Delta \rho = \rho_{{{\text{red}}}} - \rho_{{{\text{ox}}}} ,$$

where $\rho_{{{\text{red}}}}$ and $\rho_{{{\text{ox}}}}$ were the electron densities in the reduced and oxidized states, respectively. The calculations were based on the optimized geometry of the oxidized states. The changes in charge $(\Delta Q)$ were calculated as integrals of $\Delta \rho$ around selected atoms.

### Coevolution analysis

This was performed with the web-based tool CoeViz [18] integrated in the web server POLYVIEW-2D [51]. Weighted Chi-squared metric was used with 20 amino acid alphabets by sequence identity [18, 52]. The cut-off of strong co-evolution was set to 30% top scores based on the statistics of the whole co-evolution scores.

### MD simulations of oxidized and reduced human mNT in water solution

The calculations were based on the X-ray structure with PDB ID: 2QH7 [22]. H atoms were added to the heavy atoms assuming standard bond length and bond angles. The titratable residues were protonated assuming a pH of 6.0—the same as in our experiments below—using the H++ webserver [53]. The proteins were embedded in the center of a dodecahedron box with a distance of 3.0 nm or larger from the protein to the border of the box. Na^+^ and Cl^−^ ions were added to neutralize the systems and mimic our experimental ionic strengths of 100 mM NaCl, (see “Experimental procedure”, Table S5).

The protein, water and counterions were described by the AMBER99SB-ILDN [54, 55], TIP3P [56], and the Åqvist potential [57], respectively. The force field parameters of the clusters were calculated for the reduced state using MCPB.py [38] following Ref. [21] and our previous work [17] (Table 1). In particular, the restrained electrostatic potential (RESP) atomic charges [58] were calculated on **2** using the Merz–Kollman (MK) scheme [59] at the same unrestricted B3LYP/6-311++G(2d,2p) theoretical level [19, 20]. All the parameters are compatible with the AMBER99SB-ILDN force field [54, 55]. For the oxidized state, we used our previous work [17].

Periodic boundary conditions were applied. Particle mesh Ewald (PME) method [60] was used for electrostatic interaction with a cutoff of 1.2 nm. The cutoff used for van der Waals interaction was 1.2 nm. Using the LINCS algorithm [61] to constrain all of the bonds. The Nose–Hoover thermostat [62, 63] and Parrinello–Rahman barostat [64] were used to obtain the constant temperature and pressure conditions, respectively. The integration step of the MD simulation above was set to 2 fs. Each system was energy-minimized by 50,000-step steepest descent and 50,000-step conjugate gradient algorithms, respectively, then heated up to 300 K by 1-ns simulated annealing process. To pre-equilibrate the simulated systems, 50-ns isochoric-isothermal (NVT) and 50-ns isobaric-isothermal (NPT) simulations were employed orderly. Then, 1-μs production trajectories were collected at 310 K and 1 atm for data analyses. Both MD simulations equilibrate after 100 ns (see SI, Fig. S9). We select representative structures form equilibrated trajectories (that is, the last 200 ns) using a cluster analysis (see SI, Section III).

The GROMOS clustering analysis code [65] was employed to identify the representative conformations in the last 200-ns trajectories, with a cutoff of 0.11 nm of backbone. 16 and 8 representatives were obtained for the oxidized and reduced proteins, respectively.

### Codes

QM, molecular orbitals analyses and MD calculations were carried out using in Gaussian09 [47], GaussView5.0 [66], Multiwfn [67], and GROMACS 2019.4 [68, 69] software packages, respectively.

## Experimental procedure

### Proteins expression and purification

mNT protein and its H87C mutant were expressed and purified as described in Refs. [10, 45]. Briefly, the soluble part of mNT protein/H87C (residue 33–108) were inserted into the expression vector pet-28a + (Novagen). The recombinant human mNT/H87C was expressed in *Escherichia coli* BL21-RIL grown in LB supplemented with 30 μg/mL kanamycin and 34 μg/mL chloramphenicol. At an OD600 of 0.6, the cells were supplemented with 0.75 mM FeCl_3_ and the expression was activated using 0.25 mM of IPTG. Cell growth proceeded for additional 12 h at 310 K. From lysed cells, the mNT proteins were purified using Ni-agarose and size exclusion chromatography as described in Refs. [10, 22].

### Proteins reduction and in vitro stability kinetics measurement

100 μM mNT protein or H87C mutant were reduced beforehand by degassing the buffer (100 mM Bis–Tris (pH 6.0) and 100 mM NaCl) with nitrogen to remove the O_2_ from the solution. mNT/H87C proteins were then reduced using 1 mg of sodium dithionite. Immediately, the proteins (mNT and its mutant) were disposed in a 96-well plate and sealed (to prevent gas exchange). The kinetics of the [2Fe–2S] cluster release of mNT and its mutant, H87C, was monitoring by measuring the specific absorption peak of the NEET protein, at 458 nm using Synergy™ H1 plate reader, equipped with a temperature control apparatus set to at 310, 313, 315 and 318 K.

## Supplementary information

Below is the link to the electronic supplementary material.

Supplementary file1 (PDF 948 kb)

## Footnotes

1 The coevolution score of four cluster ligating amino acids with L2 and other regions of the protein is much lower than 0.7.

2 Several other functionals have been used to investigate Fe–S clusters (for instance PW91 [48] and BP86 [48, 49]).
