# Supplementary material for: The two redox states of the human NEET proteins’ [2Fe–2S] clusters
Source: J Biol Inorg Chem. 2021 Aug 28;26(7):763–74. doi: 10.1007/s00775-021-01890-8 (PMC8463382; doi:10.1007/s00775-021-01890-8)
Supplement: Supplementary file 1 — Supplementary file1 (PDF 948 kb) [file 775_2021_1890_MOESM1_ESM.pdf]

Supplementary information for

The two redox states of the human NEET proteins' [2Fe–2S] clusters

Ke Zuo[1,2,#], Henri-Baptiste Marjault[1,2,#], Kara L. Bren[3], Giulia Rossetti[4,5,6], Rachel Nechushtai[1,*], Paolo Carloni,[2,4,7,*]

[1] The Alexander Silberman Institute of Life Science, The Hebrew University of Jerusalem, Edmond J. Safra Campus at Givat Ram, 91904 Jerusalem, Israel

[2] Department of Physics, RWTH Aachen University, 52074 Aachen, Germany

[3] Department of Chemistry, University of Rochester, Rochester, NY 14627-0216, USA

[4] Computational Biomedicine, Institute of Advanced Simulation IAS-5 and Institute of Neuroscience and Medicine INM-9, Forschungszentrum Jülich GmbH, 52425 Jülich, Germany

[5] Jülich Supercomputing Center (JSC), Forschungszentrum Jülich GmbH, Germany

[6] Department of Neurology, Faculty of Medicine, RWTH Aachen University, 52074 Aachen, Germany

[7] JARA Institute: Molecular Neuroscience and Imaging, Institute of Neuroscience and Medicine INM-11, Forschungszentrum Jülich GmbH, 52425 Jülich, Germany

For Peer Review

[#]These authors contributed equally to this work.

*To whom correspondence should be addressed.
Email: rachel@mail.huji.ac.il (R. Nechushtai), p.carloni@fz-juelich.de (P. Carloni)

Section I: QM calculations

Table S1. All human NEET proteins whose structure is deposited in the PDB (15/12/2020). The residues of the two chains (A and B) for which structural information is available, as well as the presence of mutations and/or chemical modifications is also reported.

Protein	PDB ID	Res. (Å)	Sequence	Eventual mutations
mNT	2QD0[1]	1.81	A: A43-K106<br>B: D38-K106	-
	2QH7[2]	1.50	A: K42-K106<br>B: A43-E107	-
	2R13[3]	1.80	A: S30-K105	-
	3EW0[4]	1.40	A: R33-E107<br>B: R33-K104	-
	3REE[5]	1.76	A: K32-K105	-
	4EZF[6]	1.19	A: F34-T108<br>B: K42-K106	A68 Ins*
	4F1E[6]	2.40	A: K42-K106<br>B: A43-E107	D67G
	4F2C[6]	1.35	A: R40-T108<br>B: K42-K105	G66A/D67A
	4F28[6]	1.55	A: K42-K104<br>B: A43-K106	M62G
	6DE9[7]	1.95	A: T31-K105	-
NAF-1	3FNV[8]	2.10	A: D68-E134<br>B: D68-V135	C92S
	4OO7[9]	1.65	A: S69-K132<br>B: S69-V135	-

* Insertion of an alanine at position 68 in the amino acid sequence.

For Peer Review

Table S2. Spin state of the [2Fe–2S] cluster in all our QM calculations and in experiment[10].

Oxidation state	Fe_X	Fe_Y	[2Fe-2S]	Experimental values[10]
Oxidized	5/2	5/2	0	0
Reduced	5/2	2	1/2	1/2

Table S3. Effect of the protein field and thermal fluctuations on the electronic structure of the mNT [2Fe-2S] cluster: The delocalization indexes. The calculations

are based on 16 and 8 representative structures of oxidized and reduced states of the protein in aqueous solution. They are carried out at the uHF/6-311++G(2d,2p) theoretical level. The standard deviation is reported. The oxidized and reduced states values are in bold face and in italics, respectively. The difference with the calculations *in vacuo*, in parentheses, is 7% or smaller. The Fe-bound histidine is considered protonated. The Methods Section details the MD simulations and the clustering analysis obtained to identify the representative snapshots.

Fe_X–S^{Cys1}	Fe_X–S^{Cys2}	Fe_X–S_1	Fe_X–S_2	Fe_Y–S^{Cys3}	Fe_Y–N^{His}	Fe_Y–S_1	Fe_Y–S_2
**0.581±0.006**	**0.558±0.009**	**0.574±0.011**	**0.573±0.010**	**0.550±0.007**	**0.284±0.006**	**0.740±0.013**	**0.707±0.008**
**(0.014)**	**(0.003)**	**(−0.036)**	**(−0.017)**	**(0.006)**	**(0.003)**	**(−0.015)**	**(−0.029)**
0.469±0.005	*0.470±0.008*	*0.714±0.015*	*0.676±0.008*	*0.449±0.014*	*0.234±0.008*	*0.571±0.008*	*0.572±0.010*
(−0.007)	*(−0.001)*	*(0.029)*	*(−0.001)*	*(−0.008)*	*(0.000)*	*(−0.008)*	*(0.015)*

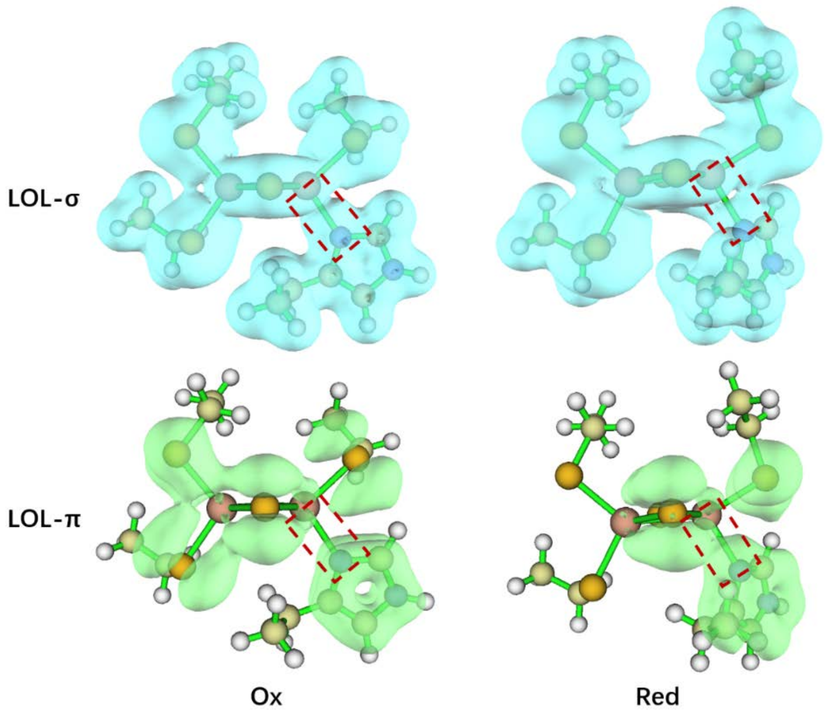

Figure S1. Histidine's σ and π bonds, analyzed in terms of localized orbital locators (LOLs). Geometry optimizations are carried out using the B(5%HF)P86[11,12]/def-TZVP functional (PDB ID: 2QH7). Both the oxidized (Ox) and reduced (Red) states are considered**.** The Fe-bound histidine is protonated. Contours of the σ bonds (light blue) and π (green) are shown at 0.20 e/Å^3. Nitrogen, iron, sulfur, carbon and hydrogen atoms are colored blue, light red, orange, light yellow and white, respectively.

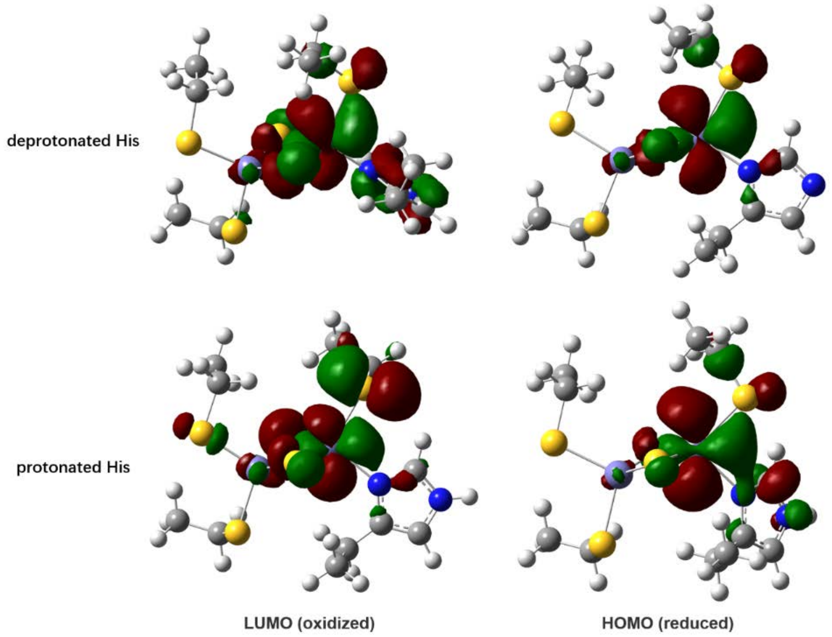

Figure S2. The LUMO of oxidized mNT [2Fe-2S] clusters (left) and the HOMO of reduced mNT [2Fe-2S] clusters (bottom), as obtained by B(5%HF)P86[11,12]/def-TZVP geometry optimizations (Model 1). The Fe-bound histidine is protonated. Contours shown at 0.02 e/Å^3. The positive and negative surfaces are depicted in green and red color, respectively.

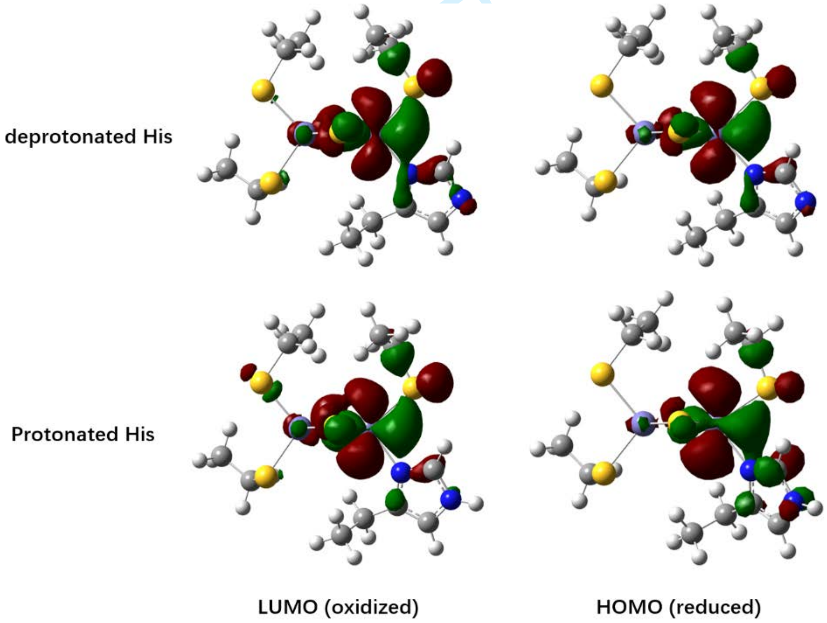

Figure S3. The LUMO of oxidized mNT [2Fe-2S] clusters (left) and the HOMO of reduced mNT [2Fe-2S] clusters (bottom) with constraints on the C$_\alpha$ atoms (See Methods: QM calculation), as obtained by B(5%HF)P86[11,12]/def-TZVP geometry optimizations (Model 1). The Fe-bound histidine is protonated. Contours shown at

0.02 e/Å^3. The positive and negative surfaces are depicted in green and red color, respectively.

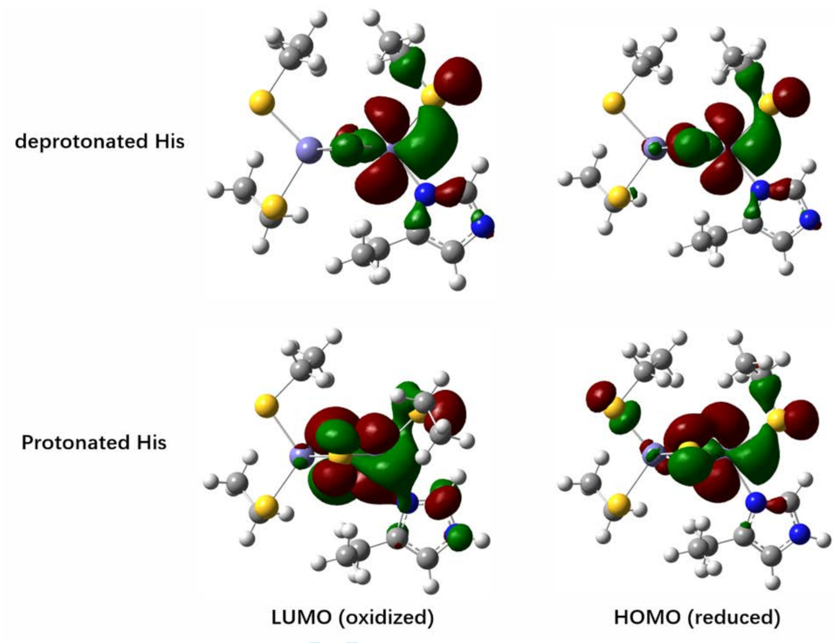

Figure S4. The LUMO of oxidized mNT [2Fe-2S] clusters (left) and the HOMO of reduced mNT [2Fe-2S] clusters (bottom), as obtained by B(5%HF)P86[11,12]/def-TZVP with DFT-D3(BJ) dispersion correction[13-15] geometry optimizations (Model 1). The Fe-bound histidine is protonated. Contours shown at 0.02 e/Å^3. The positive and negative surfaces are depicted in green and red color, respectively.

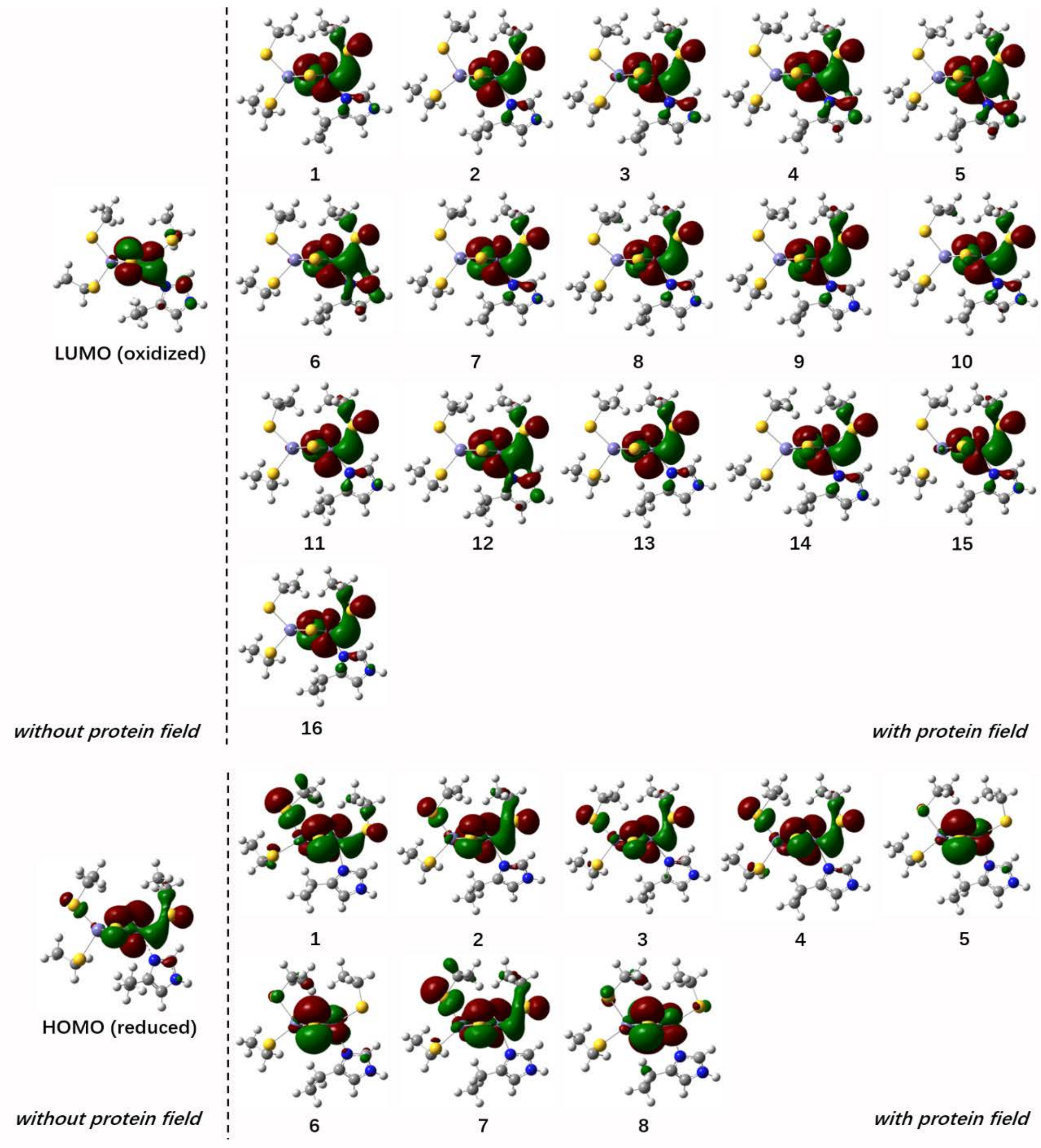

Figure S5. Effect of the protein field and thermal fluctuations on the electronic structure of the mNT [2Fe-2S] cluster: The HOMO and LUMO. (top) LUMO of the oxidized state and (bottom) the HOMO of the reduced state (model **1**), in the presence of the field of the protein/water system, simulated by MD at 310 K temperature **(**contours at 0.02 e/Å^3**).** Single point calculations with the B3LYP[16,17]/6-311++G(2d,2p) functional were carried out on the same representatives as those in Fig. S5. The single point results on model **1** *in vacuo* are also shown as reference. The Fe-bound histidine is considered protonated. In all cases, the wavefunction involves Fe_Y, with little contributions from Fe_X. The positive and negative surfaces are depicted in green and red color, respectively.

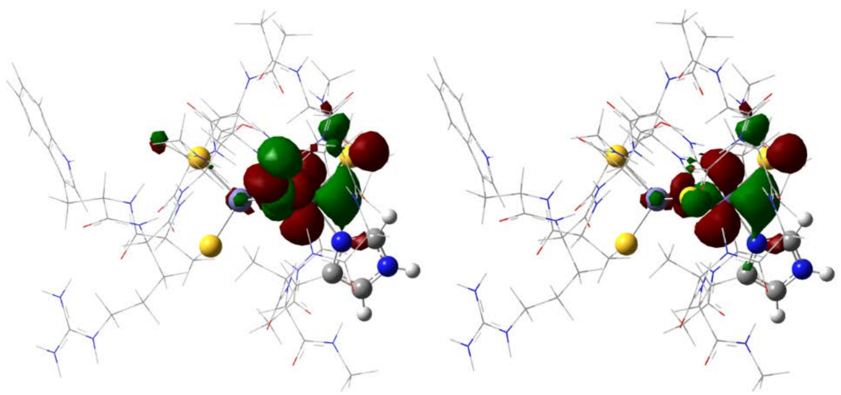

Figure S6. The LUMO of oxidized mNT [2Fe–2S] clusters (left) and the HOMO of reduced mNT [2Fe-2S] clusters (right) (model 3). They are obtained by single point calculations based on the crystal structure geometry (PDB ID: 2QH7[2]) with the B3LYP[16,17]/6-311++G(2d,2p) functional. The Fe-bound histidine is considered protonated. Contours shown at 0.02 e/Å^3. The [2Fe–2S] cluster, coordinated sulfur and imidazole moiety are represented with ball-and-stick model, and the rest parts of model **3** are shown in line. The positive and negative surfaces are depicted in green and red color, respectively.

For Peer Review

Section II: *In vitro* experiment

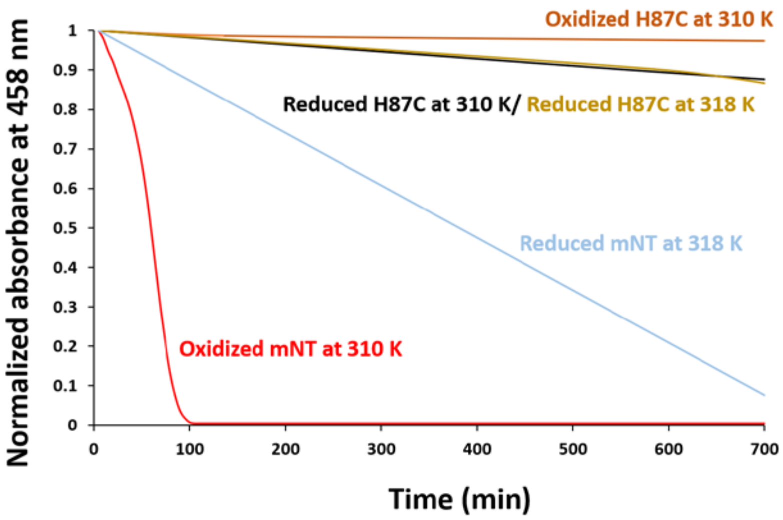

Figure S7. Lability of the reduced [2Fe–2S] clusters of wild-type and H87C human mNT at different temperatures. His87 is the iron-bound histidine in each monomer[2]. The absorbance and stability of the [2Fe–2S] clusters were monitored at their characteristic absorption peak of 458 nm[2] at pH 6.0. The clusters of the H87C mutant remain in completely or almost completely at 310 K in the oxidized and reduced states, respectively (brown and dark blue lines). Increasing the temperature does not affect at all cluster release in the reduced state. For comparison, we show here some of the results of Figure 5 main text: the wild-type protein releases the cluster at 310 K for the oxidized state, and 318 K in the reduced state (Red and light blue lines, respectively).

Peer Review

Section III: MD simulations of oxidized and reduced mNT in aqueous solution

Parametrization. Our calculations allow to build up a force field for the reduced form compatible with AMBER99SB-ILDN, as done already for the oxidized form[18]. We focus here on the mNT protein. The charges are reported in Table S4. The other parameters are listed in Table 1 in the main text.

Table S4. Top: RESP Charges of model 2, used in our MD simulations. The oxidized states charges are in bold face, the reduced states values in italics. The single point calculations were carried out with the B3LYP[16,17]/6-311++G(2d,2p) functional. Bottom: all other parameters* used in the MD simulations for the reduced form. Those for the oxidized form are reported in ref. 18. The charges on Cys1/Cys3 sulfur atoms (Figure 1) are significantly more negative than that of Cys2, possibly because the first two donor atoms form one H-bond with Cys2 and Gly85 NH groups (Figure S8), respectively while the latter, none. Indeed, the positive electrostatic potential generated by the amide hydrogen atoms stabilize relatively large negative charges

For Peer Review

	**Oxidized**				**Reduced**			
	S_1	S_2	Fe_X	Fe_Y	S_1	S_2	Fe_X	Fe_Y
	**−0.5164**	**−0.6645**	**0.8618**	**0.7015**	*−0.7678*	*−0.5862*	*0.7105*	*0.4475*
	Cys1	Cys2	Cys3	His	Cys1	Cys2	Cys3	His
$S(N_\delta)$	**−0.6045**	**−0.4662**	**−0.6677**	**−0.3252**	*−0.4815*	*−0.3446*	*−0.6010*	*−0.3211*
C_β	**0.0831**	**0.1952**	**0.2160**	**−0.5097**	*0.0275*	*0.0236*	*−0.0695*	*−0.1743*
H_β	**0.0706**	**0.0250**	**0.0318**	**0.1735**	*0.0871*	*0.0797*	*0.1299*	*0.1172*
C_α	**−0.0692**	**0.0429**	**−0.0330**	**0.1240**	*−0.4784*	*−0.3492*	*−0.4860*	*−0.4253*
H_α	**−0.0141**	**0.0766**	**0.0162**	**0.0823**	*0.2656*	*0.1587*	*0.3490*	*0.2844*
$C_{\delta 2}$				**−0.0582**				*−0.1863*
$H_{\delta 2}$				**0.1100**				*0.1325*
$N_{\varepsilon 2}$				**−0.3374**				*−0.1043*
$H_{\varepsilon 2}$				**0.3725**				*0.2299*
$C_{\varepsilon 1}$				**0.0338**				*0.0330*
$H_{\varepsilon 1}$				**0.1980**				*0.1320*
C_γ				**0.3151**				*0.2109*
C	**0.5973**	**0.5973**	**0.5973**	**0.5973**	*0.5973*	*0.5973*	*0.5973*	*0.5973*
N	**−0.4157**	**−0.4157**	**−0.4157**	**−0.4157**	*−0.4157*	*−0.4157*	*−0.4157*	*−0.4157*
H	**0.2719**	**0.2719**	**0.2719**	**0.2719**	*0.2719*	*0.2719*	*0.2719*	*0.2719*
O	**−0.5679**	**−0.5679**	**−0.5679**	**−0.5679**	*−0.5679*	*−0.5679*	*−0.5679*	*−0.5679*

Atom	σ (nm)	ε (kJ/mol)	Atom	σ (nm)	ε (kJ/mol)
Fe_X	*0.247*	*0.057*	Fe_Y	*0.251*	*0.072*

Angle	θ_{min} (°)	K_θ [kJ/(mol·rad^2)]	Angle	θ_{min} (°)	K_θ [kJ/(mol·rad^2)]
S^{Cys1}-Fe_X-S_1	*113.55*	*166.147*	S^{Cys3}-Fe_Y-S_1	*123.31*	*179.452*
S^{Cys1}-Fe_X-S_2	*112.63*	*161.753*	S^{Cys3}-Fe_Y-S_2	*121.18*	*186.941*
S^{Cys2}-Fe_X-S_1	*111.09*	*181.251*	N^{His}-Fe_Y-S_1	*114.49*	*145.687*
S^{Cys2}-Fe_X-S_2	*112.10*	*187.318*	N^{His}-Fe_Y-S_2	*104.60*	*183.301*
S^{Cys1}-Fe_X-S^{Cys2}	*102.94*	*131.378*	S^{Cys3}-Fe_Y-N^{His}	*89.10*	*96.148*
S_1-Fe_X-S_2	*104.77*	*286.311*	S_1-Fe_Y-S_2	*102.46*	*263.927*
Fe_X-S_1-Fe_Y	*76.17*	*254.136*	Fe_X-S_2-Fe_Y	*75.93*	*260.580*
C_β^{Cys1}-S^{Cys1}-Fe_X	*103.80*	*262.839*	C_β^{Cys2}-S^{Cys2}-Fe_X	*103.69*	*334.427*
C_β^{Cys3}-S^{Cys3}-Fe_Y	*104.85*	*342.586*	C_γ^{His}-N^{His}-Fe_Y	*133.52*	*328.904*
C_ε^{His}-N^{His}-Fe_Y	*116.59*	*312.587*			

***** The torsional parameters were set to 0 as in ref. 18, 19.

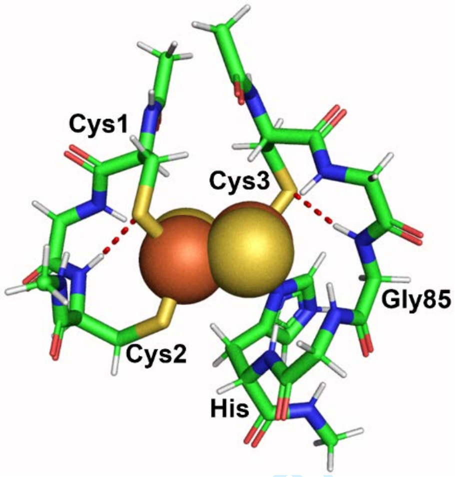

Figure S8. Hydrogen bonds (red dashes) between the main chain in the [2Fe-2S] cluster binding site and Cys1 and Cys3, respectively.

Setup. Table S5 reports some details about the simulation.

Table S5. Details of the MD simulations performed here.

Oxidation state	Charge (e)	Na^+	Cl^-	H_2O
Oxidized mNT	0	54	54	28,568
Reduced mNT	−2	54	52	28,570

Results. A plot of the RMSD as a function of simulated time (Figure S9) suggests that the system is equilibrated after 100 ns: After that time, for 900 ns, the RMSD of backbone of the mNT proteins oscillates around 0.13 ± 0.03 nm and 0.18 ± 0.02 nm for oxidized and reduced state, respectively.

Two representative structures from the two states are selected from the middle of the biggest cluster by GROMOS clustering method (see Methods)[20], respectively. The structures turn out not to differ largely (C_α atoms RMSD's = 0.5 Å, see Fig S10), except for the flexible loop at C terminal. The structures are used to identify the coevolved residues.

The flexibility of the proteins is here analyzed in terms of the T-pad analyses on the C_α atoms[21]. Larger is the T-pad value of an atom, larger is its fluctuation. We report the value for one of the two chains in Figure S11. The fluctuations are qualitatively similar for both states, with some differences in the L2 and cluster regions. We conclude that the flexibilities of the two proteins are rather similar.

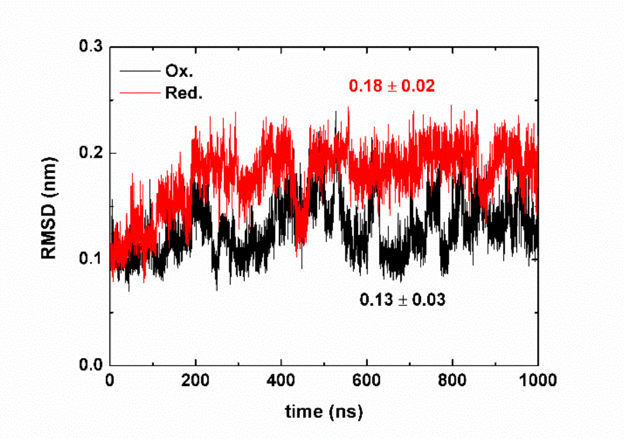

Figure S9. RMSD of backbone of the mNT in oxidized (black) and reduced (red) form versus simulation time

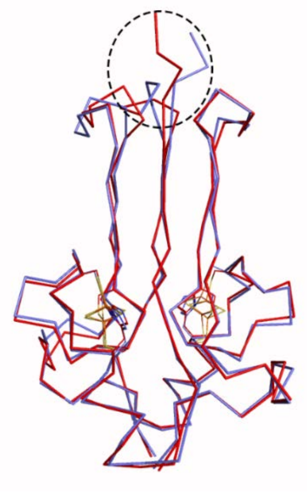

Figure S10. Superimposition of representative structures from the oxidized state (red) and reduced state (blue). Except the loop at C terminal (black circle), the backbones of mNT are superimpose well.

review

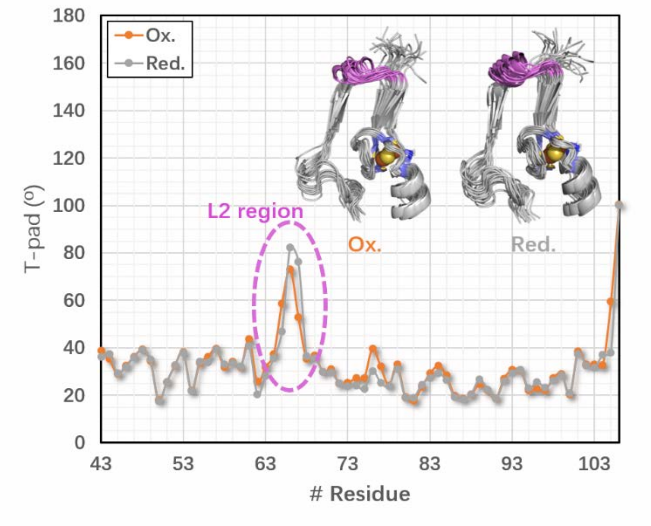

Figure S11. Comparison of the protein angular dispersion (PAD)[21] values of mNT (protomer A) in oxidized (in orange) and reduced state (in grey) based on the last 200-ns trajectory. Similar results were obtained for the other protomer simulated here.

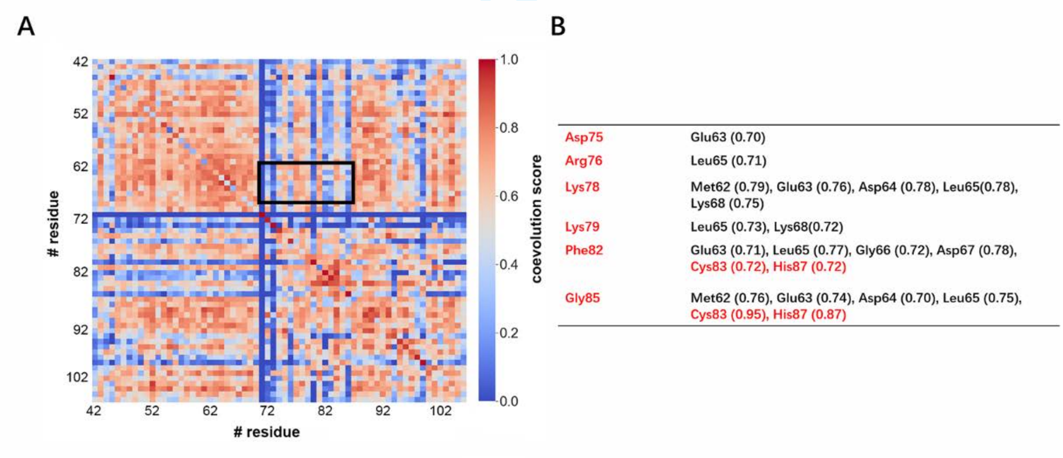

Figure S12. Allostery between [2Fe–2S] cluster area and L2 region of mNT. A The coevolution score heatmap, as obtained by CoeViz (http://polyview.cchmc.org/). The one in the oxidized state is shown in Fig. 5 in the main text. B Strongly-coevolving amino acid pairs from the cluster area and L2.

References:

1. Lin J, Zhou T, Ye K, Wang J (2007) Crystal structure of human mitoNEET reveals distince groups of iron sulfur proteins. P Natl Acad Sci USA 104(37): 14640–14645. https://doi.org/10.1073/pnas.0702426104

2. Paddock ML, Wiley SE, Axelrod HL, Cohen AE, Roy M, Abresch EC, Capraro D, Murphy AN, Nechushtai R, Dixon JE, Jennings PA (2007) MitoNEET is a uniquely folded 2Fe-2S outer mitochondrial membrane protein stabilized by pioglitazone. P Natl Acad Sci USA 104(36): 14342–14347. https://doi.org/10.1073/pnas.0707189104

3. Hou X, Liu R, Ross S, Smart EJ, Zhu H, Gong W (2007) Crystallographic studies of mitoNEET. J Biol Chem 282(46): 33242–33246. https://doi.org/10.1074/jbc.C700172200

4. Conlan AR, Paddock ML, Axelrod HL, Cohen AE, Abresch EC, Wiley S, Roy M, Nechushtai R, Jennings PA (2009) The novel 2Fe-2S outer mitochondrial protein mitoNEET displays conformational flexibility in its N-terminal cytoplasmic tethering domain. Acta Crystallogr Sect F Struct Biol Cryst Commun 65(7): 654–659. https://doi.org/10.1107/S1744309109019605

5. Arif W, Xu S, Isailovic D, Geldenhuys WJ, Carroll RT, Funk MO (2011) Complexes of the outer mitochondrial membrane protein mitoNEET with resveratrol-3-sulfate. Biochemistry 50(25): 5806–5811. https://doi.org/10.1021/bi200546s

6. Baxter EL, Zuris JA, Wang C, Axelrod HL, Cohen AE, Paddock ML, Nechushtai R, Onuchic JN, Jennings PA (2013) Allosteric control in a metalloprotein dramatically alters function. P Natl Acad Sci USA 110(3): 948–953. https://doi.org/10.1073/pnas.1208286110

7. Geldenhuys WJ, Long TE, Saralkar P, Iwasaki T, Nunez RAA, Nair RR, Konkle ME, Menze MA, Pinti MV, Hollander JM, Hazlehurst LA, Robart AR (2019) Crystal structure of the mitochondrial protein mitoNEET bound to a benze-sulfonide ligand. Commun Chem 2(1): 77. https://doi.org/10.1038/s42004-019-0172-x

8. Conlan AR, Axelrod HL, Cohen AE, Abresch EC, Zuris J, Yee D, Nechushtai R, Jennings PA, Paddock ML (2009) Crystal structure of Miner1: The redox-active 2Fe-2S protein causative in Wolfram Syndrome 2. J Mol Biol 392(1): 143–153. https://doi.org/10.1016/j.jmb.2009.06.079

9. Tamir S, Eisenberg-Domovich Y, Conlan AR, Stofleth JT, Lipper CH, Paddock ML, Mittler R, Jennings PA, Livnah O, Nechushtai R (2014) A point mutation in the [2Fe-2S] cluster binding region of the NAF-1 protein (H114C) dramatically hinders the cluster donor properties. Acta Crystallogr D Biol Crystallogr 70(6): 1572–1578. https://doi.org/10.1107/S1399004714005458

10. Dicus MM, Conlan A, Nechushtai R, Jennings PA, Paddock ML, Britt RD, Stoll S (2010) Binding of histidine in the (Cys)(3)(His)(1)-coordinated [2Fe-2S] cluster of human mitoNEET. J Am Chem Soc 132(6): 2037–2049. https://doi.org/10.1021/ja909359g

For Peer Review

11. Becke AD (1988) Density-functional exchange-energy approximation with correct asymptotic-behavior. Phys Rev A 38(6): 3098-3100. https://doi.org/10.1103/PhysRevA.38.3098

12. Perdew JP (1986) Density-functional approximation for the correlation-energy of the inhomogeneous electron-gas. Phys Rev B 33(12): 8822–8824. https://doi.org/10.1103/PhysRevB.33.8822

13. Lonsdale R, Harvey JN, Mulholland AJ (2012) Effects of dispersion in density functional based quantum mechanical/molecular mechanical calculations on cytochrome p450 catalyzed reactions. J Chem Theory Comput 8(11): 4637–4645. https://doi.org/10.1021/ct300329h

14. Grimme S (2006) Semiempirical GGA-type density functional constructed with a long-range dispersion correction. J Comput Chem 27(15): 1787–1799. https://doi.org/10.1002/jcc.20495

15. Grimme S, Antony J, Ehrlich S, Krieg H (2010) A consistent and accurate ab initio parametrization of density functional dispersion correction (DFT-D) for the 94 elements H-Pu. J Chem Phys 132(15): 154104. https://doi.org/10.1063/1.3382344

16. Stephens PJ, Devlin FJ, Chabalowski CF, Frisch MJ (1994) Ab initio calculation of vibrational absorption and circular dichroism spectra using density functional force fields. J Phys Chem 98(45): 11623–11627. https://doi.org/10.1021/j100096a001

17. Lee CT, Yang WT, Parr RG (1988) Development of the Colle-Salvetti correlation-energy formula into a functional of the electron-density. Phys Rev B 37(2): 785–789. https://doi.org/10.1103/PhysRevB.37.785

18. Pesce L, Calandrini V, Marjault HB, Lipper CH, Rossetti G, Mittler R, Jennings PA, Bauer A, Nechushtai R, Carloni P (2017) Molecular dynamics simulations of the [2Fe-2S] cluster-binding domain of NEET proteins reveal key molecular determinants that induce their cluster transfer/release. J Phys Chem B 121(47): 10648–10656. https://doi.org/10.1021/acs.jpcb.7b10584

19. Carvalho ATP, Teixeira AFS, Ramos MJ (2013) Parameters for molecular dynamics simulations of iron-sulfur proteins. J Comput Chem 34(18): 1540–1548. https://doi.org/10.1002/jcc.23287

20. Daura X, Gademann K, Jaun B, Seebach D, van Gunsteren WF, Mark AE (1999) Peptide folding: When simulation meets experiment. Angew Chem Int Edit 38 (1-2): 236–240. https://doi.org/10.1002/(SICI)1521-3773(19990115)38:1/2<236::AID-ANIE236>3.0.CO;2-M

21. Caliandro R, Rossetti G, Carloni P (2012) Local fluctuations and conformational transitions in proteins. J Chem Theory Comput 8(11): 4775–4785. https://doi.org/10.1021/ct300610y

For Peer Review
